# The Book World of Medicine and Science

**Published:** 1902-04-12

**Authors:** 


### The Book World of Medicine and Science.

Quain's Dictionary of Medicine. By various authors. Third edition, largely rewritten and revised throughout. Edited by H. Montague Murray, M.D., F.R.C.P., assisted by John Harold, M.B., B.Ch., B.A.O., and W. Cecil Bosanquet, M.A., M.D., M.R.C.P. (London: Longmans, Green and Co. 1902. Price 21s. net.)

It has rarely been the lot of a book to attain so immediately a high position as a standard work of reference as was the case with Quain's Dictionary when, nearly 20 years ago, the first edition of the work was published. The popularity of Sir Richard Quain as a consultant, the enormous pains which he had taken in the preparation of the Dictionary, and the eminence of many of the authorities by whom the articles were contributed, at once gave the book a status which it long maintained. Times change, however, and, almost more quickly still, medicine changes also, and the moment had arrived when, if the Dictionary were any longer to serve a useful purpose, a complete revision was necessary. This work has been most ably performed by Dr. Montague Murray and his coadjutors, so that while the general build of the book retains the characteristics which from the first helped to give it popularity, almost every page shows how thoroughly the task of bringing it into accord with the actual medicine of to-day has been performed. Many articles have been omitted, many have been rewritten, many new ones have been inserted, and yet it has been found possible to bring the Dictionary within the compass of a single handsome volume and to reduce the price very considerably. The great advances which have been made of late years in bacteriology have led to a general revision of the many references to this important and all-pervading topic. The article on bacteria by Dr. Welsh, which is illustrated by a very pretty, although perhaps somewhat diagrammatic, coloured plate, gives a very complete *resumé* of present knowledge on the general subject, special details concerning the various pathogenic bacteria being given under the various diseases with which they are associated. The allied subject of antitoxins is fully treated by Dr. Herbert E. Durham, who describes the different actions of antitoxic and antibacterial serums; the mode of their preparation; the mode of action of each kind of serum; their mode of administration, and many other interesting details. The article by Dr. Washbourn on serum-therapeutics to a certain extent traverses the same ground. Except in regard to diphtheria, neither writer speaks very hopefully of the clinical utility of serum treatment.

In the various articles dealing with diseases of the nervous system much will be found which is both new and interesting. Dr. Frederick W. Mott's article on Syphilis of the Brain gives a good review of this important subject. He points out that syphilis is one of the most important factors in the production of organic brain disease, and that the virus appears to act in two ways: firstly, directly upon the blood-vessels and connective tissues generally, with secondary destructive changes in the nervous tissue—a true specific inflammation; and, secondly, by direct influence upon the vitality of the neurones themselves, producing systemic degenerative changes, of which tabes dorsalis and general paralysis are by far the most common and important. The latter diseases are frequently spoken of as "parasyphilitic" or "metasyphilitic" affections. The general question of Diseases of the Blood-vessels of the Brain is dealt with by Sir William Gowers.

A short article on Dipsomania by Dr. Branthwaite, H.M. Inspector under the Inebriates Act, gives a good account of the varieties assumed by this distressing condition and the measures, legal and other, which may be required in its treatment. What he says as to the extent to which recovery may be expected is important. It is absolutely necessary that all cases should be taught the vital importance of abstinence for the rest of their lives. There is little probability, he says, of any case ever being able to drink in moderation, even after years of abstinence, without running the risk of immediately or eventually reverting to excess.

Among the many articles dealing with insanity in its various forms, a great deal that is new and interesting will be found; the article on "General Paralysis," by Dr. Percy Smith, serving well to show how pathological anatomy is pushing its way into questions of mental derangement. A thoroughly modern article on "Malarial Disease," in which the whole life-cycle of the parasite is well displayed, is written by Major Donald Ross, while other articles, such as that on gout, tend to illustrate the stationary condition of our knowledge regarding some common diseases. This new edition of "Quain's Dictionary" is well printed in small but clear type; it contains many useful illustrations, it has been brought thoroughly abreast of modern knowledge, and from the wide range of subjects with which it deals, it will probably be found even more useful than its predecessors.

Intemperance. By Professor Campbell, M.D., F.R.C.P. Ireland. (London: Burns and Oates. 1901. Pp. 35. Price 1s.)

This little book is an exhortation to all to avoid not only drunkenness but also moderate drinking. It is divided into three parts, called natural remedies, spiritual remedies, and auxiliary remedies. The whole is evidently conceived in an earnest and reverent spirit, but some of the statements are very apt to mislead, such as the following: "Between 15 and 20 years of age 18 moderate drinkers die for every three total abstainers; between 20 and 30 years of age 31 moderate drinkers die for every 10 total abstainers." This may be so, although no authority is given for the statistics, but it is obvious that the only fair comparison would be the death rate in a given number of each class. In the last part some new and interesting facts are stated, such as that the temperature of a person who has been drinking common spirits is "about two degrees lower than the healthy standard." Such a statement, if made at all, should be supported by several hundred clinical observations. Again, among the effects of the immoderate use of alcohol we read "paralysis of the lungs and heart result from injury to the pneumogastric nerve" and "paralysis of the diaphragm follows from degeneration of the phrenic nerve under the influence of alcohol." While heartily sympathising with the crusade against drunkenness, we are afraid this compilation of scriptural texts and medical statements will not convince very many and the only practical remedy suggested is the obvious one, don't drink alcohol at all.

